# Postmortem Sperm Retrieval in the Emergency Department: A Case Report and Review of Available Guidelines

**DOI:** 10.5811/cpcem.2019.9.44472

**Published:** 2019-10-21

**Authors:** Andrew R. Zinkel, Felix K. Ankel, Aaron J. Milbank, Colleen I. Casey, Jeremy J. Sundheim

**Affiliations:** * University of Minnesota Medical School, Department of Emergency Medicine, Minneapolis, Minnesota; † HealthPartners, Bloomington, Minnesota; ‡ HealthPartners Institute, Bloomington, Minnesota; § Minnesota Urology, Department of Infertility and Urology, Woodbury, Minnesota; ¶ Center for Reproductive Medicine, Department of Reproductive Endocrinology, Minneapolis, Minnesota; || HealthPartners, Department of Risk Management, Bloomington, Minnesota

## Abstract

Postmortem sperm retrieval (PMSR) requests and retrievals are increasing in the emergency department (ED) setting. Few EDs have protocols in place, and many emergency physicians (EP) lack the knowledge of how to proceed when such situations arise. We report the case of a 31-year-old male cardiac-arrest victim who expired in the ED, after which his wife requested PMSR. We review the guidelines, procedures, and issues of consent that arise with PMSR. EPs must be aware of their institution’s policies and consultant availability should a request for PMSR arise.

## INTRODUCTION

Postmortem sperm retrieval (PMSR) involves the retrieval of viable sperm from a recently deceased man for future use in assisted reproductive therapy (ART).^1^ The first case of PMSR was reported in 1980, and since then there have been several published reports of PMSR and resultant pregnancies with viable children.^2^,^3^,^4^ While both requests and retrievals in the United States have been increasing in frequency, controversy about the practice persists.^5^,^6^,^7^

Sudden unexpected accidental injury is the most common cause of death during the childbearing years and in situations in which PMSR is requested.^5^,^8^ Few emergency departments (ED) have policies for PMSR, and many emergency physicians (EP) are unaware that PMSR is even a possibility, leaving them ill-prepared to respond to these requests in an informed and timely manner.

## CASE REPORT

A 31-year-old male presented to an urban ED in pulseless cardiac arrest after his wife found him obtunded with sonorous respirations at home and began immediate cardiopulmonary resuscitation. He later expired after attempts at resuscitation were unsuccessful. Following a discussion with the decedent’s wife regarding his death, she requested we perform PMSR. The EP, having previous knowledge about PMSR in the ED, responded appropriately to the wife’s request by obtaining further history and discussing with multiple consultants.

A detailed medical history revealed that the deceased and his wife had been having difficulty conceiving a child and had begun evaluations and fertility planning at a local infertility clinic. The husband had already begun a workup for infertility with the urology group that was on call, and after consultation and further discussion it was confirmed that they follow the Weill Cornell Medicine (WCM), Department of Urology’s guidelines for consideration of requests for PMSR.^9^

Concurrent consultation was ongoing with the infertility and reproduction specialist on call from the local academic medical center, who had been seeing the wife as a patient and whose department follows the guidelines published by the Ethics Committee of the American Society for Reproductive Medicine (ASRM) on postmortem retrieval and use of gametes or embryos.^10^ Consistent with the WCM and ASRM guidelines and in discussion with the urology and infertility specialists, this case was deemed appropriate for PMSR based on presumed consent of the deceased and using the family-centered approach as described by Bahm et al. in 2013.^11^

Additional consultation with hospital legal counsel confirmed the wife had the legal rights to the decedent’s sperm. The medical examiner granted permission to perform the procedure to harvest the sperm from the body of the deceased as an autopsy was planned. Ice packs were placed in the groin and the body was transferred to the hospital morgue. The next morning the urologist consented the wife and the mother of the deceased for the harvesting procedure, and approximately 15 hours following the time of death sperm was successfully harvested and transported by an embryology technician to the academic medical center’s cryopreservation laboratory without complication.

## DISCUSSION

Requests for PMSR are rare but increasing in frequency.^5^,^6^ As in this case, they tend to occur as a request from a spouse after a sudden and unexpected death in an otherwise healthy young person. EPs are more likely than other providers to receive a request for PMSR and must be prepared to respond to this request in an informed manner. The evaluation of the eligibility of a request for PMSR involves many issues that include legal, medical, logistical, ethical, and consent issues. Many of these issues can be made clearer when following an appropriate guideline. Currently there are no national guidelines, regulations, or restrictions related to PMSR.^9^ This leaves institutional guidelines, medical society guidelines, and state laws, which vary considerably on the topic of PMSR, to guide decision-making.

### Available Guidelines

The first guideline on PMSR was published in 2003 when Tash et al. described the effects of instituting an exclusionary institutional guideline, which had been developed at the WCM by a cross-functional panel of experts that included the urology, reproduction and infertility, law, psychology, and ethics departments.^12^ Of the 22 families that sought PMSR after implementation of the guideline only four men were found to be candidates and underwent successful PMSR. Of these four cases, only one wife (of the deceased) attempted in vitro fertilization after an appropriate bereavement period, but no pregnancy occurred as a result.^12^

The number of institutions with guidelines now in place for PMSR has also continued to rise. Batzer et al. suggested 10 core elements for the development of a protocol for PMSR and recommended considering the interests of five key stakeholders to include the deceased, the requesting party the resultant child the physician, and society.^1^

In 2013 Bahm et al. contacted 40 institutions and evaluated nine full PMSR protocols.^11^ They identified six major components present in most protocols and described two major approaches to policy structure: an institutional, limited-role approach, and a more family-centered approach.^11^ In the limited-role approach, institutions require written consent before the death of the donor, specification of who can receive the sperm, and relinquishment of all responsibility after sperm retrieval. In the family-centered approach, the recipient of the sperm is allowed to use substituted judgment in the absence of written consent, but requires a period of psychological counseling before the sperm can be used in ART. The protocols evaluated varied along a spectrum between these two approaches.^11^

### Legal Issues

Two major legal issues may arise in PMSR regarding the retrieval and/or the use of tissue in posthumous reproduction: 1) Does the wife have the legal right to the decedent’s sperm for use in reproduction; and 2) are children of the deceased legally recognized as offspring?^10^ More recent case law has raised the debate of whether or not treating tissue retrieved from PMSR similarly to organ donation tissue under the Uniform Anatomical Gift Act of 2006 is appropriate.^13^ Jurisdictions and case law vary in regard to the interpretation of these laws and the answers to these questions. In our case, legal consultation confirmed the wife’s right to the decedent’s sperm. However, our institution did not have a guideline in place. It was quickly confirmed that our urology group followed the WCM guidelines, while the reproductive and infertility group followed the ASRM guidelines.^9^,^10^

### Ethical Issues

According to both the WCM and ASRM guidelines requests for PMSR should only be considered when requested by the wife of the deceased and she should be the only person for whom the sperm could be used for procreation. Requests from other family members or next-of-kin should not be considered.^9^,^10^

Issues regarding consent of the deceased, or lack thereof, are the most common reason not to proceed with a request for PMSR. The gold standard would be to have expressed written consent from the deceased to proceed with PMSR and ART in the case of his death. This is a rare occurrence, but does occur in instances of planned ART in which the consent forms specifically dictate the disposition of obtained gametes after the death of the individual from whom they were contributed.^10^ The goal of presumed consent in PMSR is to ensure that the deceased had previously expressed a desire to conceive or was already attempting to conceive with his wife. Providers should consider “stated, written, or acted on wishes prior to death” when making decisions regarding PMSR.^9^

**CPC-EM Capsule**

What do we already know about this clinical entity?

*Postmortem sperm retrieval (PMSR) requests are increasing in frequency in the emergency department (ED) setting, and few EDs and physicians are prepared should such a request arise*.

What makes this presentation of disease reportable?

*A descriptive case report on PMSR based on available guidelines has not been presented in the emergency medicine literature before*.

What is the major learning point?

*It is within the emergency physician’s (EP) scope of practice to make an informed decision in response to a request for PMSR*.

How might this improve emergency medicine practice?

*It educates EPs on the issues related to PMSR in the ED, appropriate consultants to involve, and how to find available guidelines*.

### Logistical Issues

In cases that will require an autopsy, permission to perform PMSR should be obtained from the medical examiner. Additionally, the medical history of the deceased should confirm that there are no medical conditions that would have prevented sperm production and there should be no evidence of communicable disease, which may require additional testing.^9^ Several procedural methods can be used to perform PMSR, with the least-invasive methods being the most preferred.^12^ The WCM guidelines suggest retrieval be performed within 24 hours of death, while other sources cite up to 36 hours as appropriate to retrieve viable sperm.^14^

Transfer of the body to the morgue after expiration and harvesting of the sperm in less than 24 hours results in a high likelihood of viable sperm (86%, with a mean time to retrieval of 20.4 hours after death) and chances similar to ART of a resultant pregnancy.^14^ Discussions with consultants can be done in a less urgent manner while the body is in the morgue, as in any other death in the ED, and will not use further nursing or departmental resources after this point. PMSR requires a cryopreservation facility to be available and nearby for immediate processing of the specimen.

The WCM and ASRM guidelines agree that PMSR programs are not ethically obligated to perform ART even though they may allow for PMSR to occur at their institution. They are also in agreement that PMSR programs should require a period of time for the normal bereavement process and psychological counseling to occur before the wife has the ability to use the sperm in ART.^9^,^10^ The WCM guideline specifically requires a one-year quarantine period, which is used to evaluate the wife’s family support system and understanding of the implications of raising a child in the absence of the biological father.^9^ The observations from WCM indicate that the majority of women reconsider their decision and do not proceed with ART after they have had time to grieve and receive appropriate counseling.^9^

## CONCLUSION

It is within the EPs scope of practice to make an informed decision in response to a request for PMSR. EPs must understand the issues surrounding consent, ethics, logistics, legality, and the various institutional practice guidelines and consultants available to them should such a request arise.
